# Genomic ancestry and the social pathways leading to major depression in adulthood: the mediating effect of socioeconomic position and discrimination

**DOI:** 10.1186/s12888-016-1015-2

**Published:** 2016-09-05

**Authors:** Christian Loret de Mola, Fernando Pires Hartwig, Helen Gonçalves, Luciana de Avila Quevedo, Ricardo Pinheiro, Denise Petrucci Gigante, Janaína Vieira dos Santos Motta, Alexandre C. Pereira, Fernando C. Barros, Bernardo Lessa Horta

**Affiliations:** 1 Postgraduate Program in Epidemiology, Federal University of Pelotas, Rua Marechal Deodoro, 1160 - 3° Piso, Bairro Centro. Cep: 96020-220, Caixa Postal 464, Pelotas, RS Brazil; 2 Health and Behavior Postgraduate Program, Universidade Católica de Pelotas – UCPEL, Rua Gonçalves Chaves, 377 - sala 411, prédio C. CEP: 96015-560, Pelotas, RS Brazil; 3 Heart Institute, University of São Paulo Medical School, Av. Dr. Arnaldo, 455 - Cerqueira César - CEP: 01246903, São Paulo, SP Brazil

**Keywords:** Major depression, Genomic ancestry, Socioeconomic position, Discrimination, Brazil, Cohort

## Abstract

**Background:**

Evidence suggests that there is an association between ethnicity/skin color and depression; however, many contextual and individual variables, like sense of discrimination and socioeconomic position (SEP), might influence the direction of this association. We assessed the association between African ancestry and major depression among young adults that have been followed-up since birth in a Southern Brazilian city, and the mediating effect of SEP and discrimination.

**Methods:**

In 1982, all hospital deliveries in Pelotas (Southern Brazil) were identified; liveborns were examined and their mothers interviewed (*n* = 5914). In 2012–13, at 30 years of age, we used the Mini International Neuropsychiatric Interview (MINI) for major depression diagnosis. In addition, DNA samples were genotyped for approximately 2.5 million single nucleotide polymorphisms (SNPs) using Illumina (CA, USA) HumanOmni2.5-8v1 array. Genomic ancestry estimation was based on approximately 370 000 single nucleotide polymorphisms (SNPs) mutually available for the Pelotas cohort and selected samples (used as reference panels) of the HapMap and Human Genome Diversity (HGDP). We estimated prevalence ratios (PR) using Poisson regression models and evaluated the association between percentage of African ancestry and major depression. We used G-computation for mediation analysis.

**Results:**

At 30 years, 3576 individuals were evaluated for major depression (prevalence = 7.9 %). Only individuals in the highest SEP, who had a percentage of African ancestry between >5–30 % and >30 % had a prevalence of major depression 2.16 (PR = 2.16 95 % CI [1.05–4.45]) and 2.74 (PR = 2.74 95 % CI [1.06–7.06]) times higher, than those with 5 % or less, respectively. Among these subjects, sense of discrimination by skin color, captured 84 % of the association between African ancestry and major depression.

**Conclusion:**

SEP is an important effect modifier of the positive association between African ancestry and major depression. In addition, this association is predominantly mediated by the sense of feeling discriminated by skin color.

**Electronic supplementary material:**

The online version of this article (doi:10.1186/s12888-016-1015-2) contains supplementary material, which is available to authorized users.

## Background

It has been suggested that ethnicity and skin color are associated with the development of depression, and factors such as discrimination and/or socioeconomic position (SEP) could be potential mediators or effect modifiers [1–5]. In the United States, it has been reported that depression is more prevalent among Caucasian/white individuals than African Americans or other ethnic groups [6–9]. However, in Brazil, some studies have reported a higher prevalence of depression among “morenos”, “mulattos” and poor black women of African ascendency [1], whereas others have observed a higher risk of depression in individuals suffering from racial discrimination [10].

Product of different social structure patterns; factors like, perception of racial/ethnic discrimination and lack of sense of belonging, might be part of the casual pathway between ethnicity/skin color and depression, especially in settings where there is little or no ethnic social support [2, 5, 10–13].

Populations like Brazil, and other South American countries, have a great amount of individuals of African ascendency. However, it is important to consider that these are genetically admixed populations, product of post-Columbian admixture between Amerindians, Europeans colonizers or immigrants, and African slaves and consequently individuals of black or white skin color could have a considerable amount of African and European ancestry. Interestingly, Brazil was the destiny of nearly 40 % of the African diaspora, receiving seven times more slaves than the United States. In addition, Brazil is a complex and diverse population, where self-report of race/skin color is subjective. In places like Bahia (northeastern Brazil), you can find a large amount of individuals of African ancestry with black skin color, in this places report of race/skin color might be different than others, like southern Brazil, where individuals of African ancestry or black skin color are a minority and the population is less admixed. A previous report from the EPIGEN-BRAZIL, using data from three Brazilian cohorts, each one from different regions of the country (south, southeast and northeast Brazil), showed that these populations are genetically admixed at different levels, and that the patterns of association between self-reported skin color and genomic ancestry differ by site, probably because of the admixture level. The 1982 Pelotas birth cohort is part of this consortium, and in this population, the proportion of African ancestry was 6.6 % (95 % CI 3.8–16.3 %) [14].

It is important to mention that Brazil never had segregation laws, different from the United States and South Africa. Therefore, the Brazilian population is more mixed, and its ethnoracial classification is more complex and fluid than in those countries where segregation was imposed by law [15]. In highly genetically admixed populations, such as in Brazil, personal information on ethnicity might not provide the same robust estimations as in less diverse populations [16]. Product of the incorporation of various social cultures in Brazil, the genotypic and phenotypic characteristics of these populations have been added to the native population, probably making physical appearance characteristics such as skin color a poor indicator of the geographical origin of a Brazilian individual’s ancestors.

Therefore, self-perception of skin color/race could be biased by the context in which a person live, especially in Brazil, where different regions of the country present different levels of admixture. This could also be important in terms of discrimination; individuals of certain race minorities, in less admixed populations, could be more prone to suffer different kinds of discrimination, and they could feel a lack of sense of belonging, which could at the same time influence in their self-declaration of race/skin color. Populations from southern Brazil, like our sample, have a low level of admixture.

To our knowledge, no previous study has evaluated pathways linking mental health and genomic ancestry. Furthermore, it is relevant to explore which aspects of the context in which a person lives could interfere with these associations [17].

This study was aimed at assessing the association between African ancestry and major depression in a Southern Brazilian population of young adults that have been prospectively followed since birth. We also explored the role of SEP and/or discrimination as mediators or effect modifiers of this association. By using genomic ancestry, we are assessing a social determinant like skin color/race, with a different approach, not just the classic self-report, used in most studies. It is not our objective to establish if certain specific genetic variations, used to assess ancestry, are associated with depression, but to use them as a whole, as a proxy of skin color/race.

## Methods

In 1982, all maternity hospitals in Pelotas, a southern Brazilian city, were visited daily and all newborns whose families lived in the urban area of the city were examined and their mothers interviewed (*n* = 5914) [18].

In 2004–2005, at the mean age of 22.8 years, we tried to follow the whole cohort; individuals were interviewed at home and invited to visit the clinic for blood collection. DNA was extracted (*n* = 3736) and stored at -70 °C. Later, these samples were genotyped for approximately 2.5 million single nucleotide polymorphisms (SNPs) using Illumina (CA, USA) HumanOmni2.5-8v1 array. Genomic ancestry estimation was based on approximately 370,000 SNPs mutually available for the Pelotas cohort and selected samples (used as reference panels) of the HapMap and Human Genome Diversity (HGDP). ADMIXTURE was used for genomic ancestry estimation [19]. The selected samples were 266 Africans (Yoruba and Luyha), 262 Europeans (American and Italian), 77 admixed Mexican Americans, 83 African Americans (all these from HapMap), as well as 93 Native Americans (from the Human Genome Diversity Project). At the end, we estimated the proportions of European, African American and Native American ancestries at the individual level. In Pelotas, with few exceptions, cohort members were unrelated and have low consanguinity [20]. Further details on genotyping techniques and results on genomic ancestry association with self-reported skin color have been published elsewhere [14, 20].

From June 2012 to February 2013, at a mean age of 30.2 years, we followed 3701 individuals of the original cohort. [21] In this visit, a psychological interview was carried out, including a diagnostic interview for major depression using the Mini-International Psychiatric Interview (MINI) V5.0 validated for Brazil [22]. We considered a person as having major depression if he or she presented an episode of major depression during the last two weeks and did not report a manic/hypomanic episode during life, according to the MINI.

Because African and European ancestries were highly correlated (Pearson’s correlation coefficient = -0.97), and Native American proportions were low, we decided to use African ancestry only. In the data analysis, initially, the proportion of African ancestry was expressed in deciles. After that, this variable was recorded into three categories. The first category represented the first four deciles (proportion of African ancestry ranging from 0 to 5 %) the second category represented the deciles five to eight (proportion of African ancestry ranging from more than 5 to 30 %) and the third category the deciles nine and ten (proportion of African ancestry ranging from more than 30 to 90 %). These deciles were joined together based on mean similarities of African ancestry. Because our sample have a skewed distribution of African ancestry, we preferred to use this variable, which represents three homogeneous groups of individuals, in terms of ancestry.

At the mean age of 30.2 years, we measured three different socioeconomic variables. Achieved schooling, the highest school grade successfully completed by the individual; household assets index score, based on the individuals and family belongings and possessions, this was a continuous variable, which was then categorized in three (high, middle, and low); and family income: total income, in Brazilian reais, earned by family members in the last month.

We used exploratory factorial analysis to create a single variable with these three variables of SEP. In addition, this new SEP variable was categorized into three tertiles, the first tertile represented the lowest SEP and the third the highest.

We assessed sense of discrimination by skin color; religion or beliefs; and/or for being rich or poor. Each type of discrimination was treated separately as a single variable. Therefore, an individual who referred feeling discriminated because of the skin color, religion or beliefs; and/or for being rich or poor, was considered as a victim of skin color discrimination, religious discrimination and/or socioeconomic discrimination, respectively.

Details on socioeconomic variables and discrimination measures can be found in our Additional file 1.

At 23 years, cohort members were asked to self-declare their skin color as white, black, mulatto, yellow, indigenous or moreno. For analysis purposes, we merged the last four categories (mulatto, yellow, indigenous and Moreno) into one named “pardo”.

We used Chi-square test and Fisher exact test to compare proportions, and Kruskal-Wallis test to evaluate differences between medians. Poisson regression with robust adjustment of the variance was used to estimate prevalence ratios (PR) [23]. We also tested if SEP, as well as the sense of feeling discriminated, modified the association between African ancestry and major depression in a Poisson regression model adjusted for sex. We calculated 95 % confidence intervals (95 % CI) for proportions and PR, and interquartile ranges (IQR) for medians.

We assessed the mediating effect of SEP and discrimination on the association between African ancestry and major depression using G-computation [24] to estimate the natural direct effect (NDE) and natural indirect effect (NIE) of African ancestry on major depression. The NDE represents the effect of the exposure (African ancestry) on the outcome (major depression) that is not captured by the mediator (SEP or discrimination), while the NIE estimates the effect that is captured. Therefore, the sum of the NDE and NIE would represent the total effect, and the quotient of dividing the NIE by the total effect would represent the percentage of the effect that is captured by a mediator [25]. In our mediation analysis, sex was considered a base confounder, and SEP at 30 years as a post confounder when analyzing the mediating effect of discrimination.

## Results

We applied the MINI interview to 3576 individuals. Prevalence of major depression was 7.9 %. The proportions of African and European ancestry were 6.6 % (95 % CI 3.8–16.3 %) and 85.3 % (72.8–91.0 %), respectively. Since the Pearson’s correlation coefficient between African and European ancestry was -0.97, we only used African ancestry in our analyses. The follow-up rate at 30 years was higher among females, those whose mothers had between 5 to 8 years of schooling, self-reported as non-white and whose family income ranged from 1–6 minimum wages. No follow-up differences were found from 23 to 30 years in terms of cohort members self-reported skin color. Among those subjects who were interviewed at 30 years, 96.6 % of them participated in the psychological evaluation, and no differences were found when compared to those who did not answer the mental health questionnaire (Additional file 2: Table S1).

Table 1 shows that 52 % of the individuals were females, 75 % self-reported as white, mean achieved schooling was 11 years, and median income in the month before the interview was 2200 Brazilian Reais (1.8 Reais = 1USD). In addition, 5.8 % reported feeling discriminated because of their race/skin color, 7.4 % for their religion or beliefs, and 6.9 % for been poor or rich; 15.6 % of the individuals reported feeling discriminated for at least one of these reasons. Of those who reported feeling discriminated because of their skin color, 59.5 % self-reported as black, 14.7 % as pardo and 25.8 % as white, in addition, 68.2 % had more than 30 % of African ancestry, 19 % had between 5 and 30 %, and 12.9 % less than 5 %.

**Table 1:** Sociodemographic and biological description of the studied population

	N	Mean/Median (SD/IQR)^a^	Prevalence
Female	1859		51.9 %
Skin color white	2455		75.1 %
Income at 30 years	3402	2200 (1320–3900)	
Years of schooling at 30 years	3555	11.3 (4.1)	
Assets index 30 years			
High	1859		65.8 %
Middle	849		30.3 %
Low	109		3.9 %
Discrimination by race, religion and/or SEP	459		15.6 %
African ancestry			
0–5 %	1186	3.4 % (2.5–4.3 %)	40 %
> 5–30 %	1160	8.7 % (6.7–12.9 %)	40 %
> 30–90 %	599	50 % (37.6–61.6 %)	20 %
Major Depression	284		7.9 %

^a^ *SD* standard deviation, *IQR* interquartile range

African ancestry was negatively associated with socioeconomic status. Moreover, African ancestry was positively associated with discrimination. Major depression was more common among females, individuals that felt discriminated, and among those in the lowest SEP. No association was found between skin color and major depression (Table 2).

**Table 2:** Distribution of sociodemographic characteristic and discrimination, according to percentage of African ancestry and major depression

		Total N	African ancestry				Major depression	
			0–5 %	>5–30 %	>30–90 %	p	Prevalence	p
Sex	Male	1717	40.7 %	39.4 %	19.9 %	0.797	4.2 %	<0.001
	Female	1858	39.8 %	39.3 %	20.8 %		11.2 %	
Self-reported skin color	Black	516	0.8 %	6.7 %	92.5 %	<0.001	9.3 %	0.193
	Pardo	297	10.4 %	46.5 %	43.1 %		9.4 %	
	White	2455	52.5 %	45.6 %	2.0 %		7.4 %	
Discrimination ^a^	No	2478	42.2 %	40.1 %	17.7 %	<0.001	6.7 %	<0.001
	Yes	459	29.0 %	35.9 %	35.1 %		14.2 %	
Skin color discrimination	No	2833	41.9 %	40.8 %	17.3 %	<0.001	7.4 %	<0.001
	Yes	179	12.9 %	18.9 %	68.2 %		16.9 %	
Religious discrimination	No	2704	40.6 %	39.4 %	20.0 %	0.174	7.7 %	0.052
	Yes	230	35.2 %	40.4 %	24.4 %		11.3 %	
Socioeconomic discrimination	No	2724	41.2 %	39.4 %	19.4 %	<0.001	7.4 %	<0.001
	Yes	209	27.3 %	40.2 %	32.5 %		15.3 %	
Schooling at 30 years	0–4	211	28.3 %	41.1 %	30.6 %	<0.001^b^	15.6 %	<0.001^b^
	5–8	707	29.3 %	42.5 %	28.3 %		11.3 %	
	9–11	1069	35.1 %	41.9 %	23.0 %		6.9 %	
	≥12	1568	50.5 %	36.0 %	13.5 %		5.9 %	
Assets index at 30 years	High	1859	47.2 %	38.6 %	14.2 %	<0.001^b^	5.8 %	<0.001^b^
	Middle	849	26.7 %	43.5 %	29.8 %		11.1 %	
	Low	109	17.8 %	43.3 %	38.9 %		15.6 %	
Income at 30 years (tertiles)	1st	1131	31.7 %	40.5 %	27.7 %	<0.001^b^	10.0 %	<0.001^b^
	2nd	1121	37.9 %	41.4 %	20.7 %		7.3 %	
	3rd	1150	52.5 %	35.5 %	12.0 %		4.7 %	

^a^Any kind of discrimination

^b^Trend *p*-value, all other *p*-values are from chi-squared test for heterogeneity

In multivariable analysis, an increase of one percentage point in African ancestry increased the risk of depression in 0.6 % (PR = 1.006 95 % CI [1.00–1.01]). However, as shown in Table 3, SEP modified the association between genomic ancestry and major depression (interaction term *p* = 0.017). Only in the highest tertile of SEP, prevalence of major depression was higher in individuals with a proportion of African ancestry between >5–30 % (PR = 2.16 95 % CI [1.05–4.45]) and >30–90 % (PR = 2.74 95 % CI [1.06–7.06]), whereas no association between African ancestry and major depression was observed among those in the first and second tertiles of SEP. Similarly, in a sub analysis that included only those individuals who self-reported as white, we found the same pattern of association, major depression was 2.73 times higher (PR = 2.73 95 % CI 1.27–5.82) and 6.49 times higher (PR = 6.49 95 % CI 0.93–45.1) among those subjects with a proportion of African ancestry between >5–30 % and >30–90 %, respectively. In addition, prevalence of perceived skin color discrimination was higher among self-reported white individuals, with higher African ancestry, however differences were not statistically significant (*p* = 0.275).

**Table 3:** Multivariable regression models for major depression and African ancestry by tertiles of socioeconomic position (SEP)

	N	African ancestry median [IQR]	African ancestry	PR (95 % CI)^a^	*p*-value^b^
*SEP*					
*1* ^*st*^ *tertile*	756	0.09 [0.05–0.30]	0–5 %	1	0.293
			>5 %–30 %	0.98 (0.60–1.59)	
			>30 %–90 %	0.74 (0.42–1.31)	
*2* ^*nd*^ *tertile*	767	0.07 [0.04–0.18]	0–5 %	1	0.997
			>5 %–30 %	1.02 (0.56–1.87)	
			>30 %–90 %	0.99 (0.48–2.04)	
*3* ^*rd*^ *tertile*	724	0.05 [0.03–0.09]	0–5 %	1	0.009
			>5 %–30 %	2.16 (1.05–4.45)	
			>30 %–90 %	2.74 (1.06–7.06)	

*PR* prevalence ratio, *CI* confidence interval, *IQR* interquartile range

^a^Multivariable models using major depression as the outcome and African ancestry as main exposure are adjusted for sex

^b^Trend *p*-value for African ancestry in each tertile of SEP

We did not find an association between self-reported skin color and depression, even when joining different categories of self-report. However, individuals who self-reported as black reported a higher proportion of discrimination, by race, religion and been rich or poor, compared to whites, same as for Pardo (Additional file 3: Table S2).

**Fig. 1:**
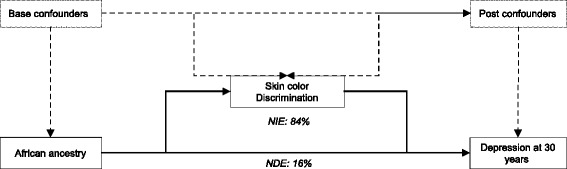
Direct Acyclic Graph of the effect of african ancestry on major depression at 30 years. Natural Indirect effect (NIE) shows that 84 % of the total effect of ancestry on depression at 30 years is mediated by racial sense of discrimination and only 16 % through a Natural Direct effect (NDE). Estimates were adjusted for base confounders: sex; and post confounders: achieved schooling and income at 30 years

Mediation analysis among those in the highest tertile of SEP, showed that sense of discrimination by skin color captured 83.9 % of the association between African ancestry and major depression (Fig. 1 and Additional file 4: Table S3).

## Discussion

### Main findings

In a cohort that has been prospectively followed since birth, in a southern Brazilian city, we found that socioeconomic status modified the association between genomic ancestry and major depression in early adulthood. Among those individuals in the highest tertile of SEP, African ancestry was associated with a higher prevalence of major depression and discrimination captured most of this association. The pattern of association was similar when we analyzed only individuals who self-reported as white; in addition, among these individuals, discrimination appeared to be higher in those with higher African ascendance, however, few had high African ancestry (*n* = 43), consequently our small sample size precluded any conclusions, for this sub analysis.

In our sample, the association between African ancestry and major depression was only present in high socioeconomic contexts, where median African ancestry was approximately 5 %, lower than in other categories of SEP. In addition, people with higher African ancestry had lower SEP. Consequently, individuals in the highest levels of SEP, with higher African ancestry could have a lack of sense of belonging to the group, and probably a higher sense of discrimination. It has been shown that in social contexts where people feel as a minority (racial or ethnic) and have no social support, mental health problems, especially depression, are more common [5, 9, 26]. In addition, probably intra-group discrimination is higher in individuals of high SEP; however, this dimension of discrimination was not evaluated.

It has been shown that immigrants and ethnic minorities have higher rates of mental health problems. For example, studies evaluating international migrants in Europe have shown a higher prevalence of depression and other affective disorders among these individuals [27, 28] as well as for ethnic minorities living within neighborhoods of individuals from other ethnic groups of the same nationality [9, 26].

Lack of social support and sense of belonging to the group, can also have deteriorating effects in social and academic life. Winter-Colins [29] showed that sense of belonging was positively associated with the individuals’ job satisfaction. In addition, it has been reported that school achievement is impaired in adolescents and children with lack of sense of relatedness, belonging and interpersonal support. [30, 31] Indeed, sense of belonging has been associated with different social and environmental factors, which are ultimately related to social and psychological functioning [32].

This all suggests that living in a social environment where someone feels as a minority, independently of other factors, could predispose to depression [2, 5, 10]. In our sample, individuals from high SEP with high African ancestry could be suffering from this same lack of sense of belonging, and combined with a poor social support, this could create an environment where discrimination could be a trigger for the development of depression.

Moreover, inequalities in mental health morbidity between and within ethnic minorities are linked to SEP, and discrimination could be an important factor in the casual pathway [33]. In a study carried out in Salvador [10] – a Brazilian city with a proportion of African ancestry higher than that in Pelotas [14, 20] – the authors did not detect any association between skin color and major depression, however, perception of racial discrimination was strongly associated to major depression. On the other hand, Almeida-Filho [1] found, in another sample from Salvador, that depression was higher in non-white women from poor working classes, specially “mulatto” and black women. In our study, African ancestry was associated with depression among individuals with high SEP, these differences between our results and the ones from Salvador could be explained by the context in which individuals of these populations live and type of discrimination suffered.

The mediation analysis suggests that almost all the association between African ancestry and major depression is captured by sense of discrimination, in high socioeconomic settings. Discrimination has been reported as a risk factor for the development of depression. Ayalon [2] and Santana [10] reported higher prevalence of depression in individuals suffering from discrimination. We also found an association between discrimination and depression, but we did not find an interaction between ancestry and discrimination.

### Strengthens and Limitations

Discrimination is a complex concept that includes several different types, conditions and scenarios that have not been explored in this study, including age, gender, or even internalized discrimination, intra-group, institutional, and structural discrimination [11, 13].

Probably intra-group discrimination is higher among individuals of high SEP, and that is the reason why it was a mediator of this association only in this group. Because of its complex nature, discrimination, was treated in this article as the sense of feeling discriminated, we were not able to explore all of its dimensions, however we consider that our findings are strong and show good evidence that skin color discrimination mediates the association between African ancestry and major depression.

In our sample, self-reported skin color was not associated with major depression, only African ancestry. Similarly Santana et al. [10] found no association in Salvador, however, they did not assessed the effect modification of discrimination nor SEP, and Almeida-Filho et al. [1], found an association only among women from poor working classes, specially “mulatto” and black. The Pelotas sample is less admixed, than those from Salvador and fewer people self-reported as Black or Pardo [14], consequently, the power to test the association between self-reported skin color and depression could have been low in Pelotas. Nonetheless, as presented in Additional file 3: Table S2, we tested this association grouping our self-reported skin color variable, in different ways, and found no association.

This is the first study to our knowledge to explore possible casual pathways in the association between genomic ancestry and major depression during adulthood in a middle-income setting. Our study has the strength of using a quantitative measurement of ancestry. We used approximately 370 000 SNPs to estimate the genomic ancestry of each individual, something that only a few have accomplished to date (Additional file 5).

## Conclusion

In this low admixed sample, we have found that individuals of high SEP with higher African ancestry are more likely to be discriminated and therefore are at a higher risk of presenting major depression during early adulthood. Therefore, tackling discrimination in this specific at risk population could significantly reduce the prevalence of major depression.

However, since this is the first time this kind of results have been presented for a population with low admixture levels, and other Brazilian or non-Brazilian populations with higher levels of admixture could present different social structures, other studies should focus on validating these findings.

## Additional files

Additional file 1: Details for socioeconomic and discrimination variables. Here we present more details on how socioeconomic variables, achieved schooling, family income, and household assets index score, were collected and stratified during the last follow-up (30 years). In addition, we explain how this were joined together using a factorial analysis. Details, labels and other information regarding Database “data afr dep bmc.csv”. We included the file “data afr dep bmc.csv”, in order to follow the norms of availability of data and materials. In this section of our Additional file 1, we have included details on the variables in this database. This includes categorization of the variables, labels, and other relevant information. (DOCX 15 kb) Additional file 2: Table S1. Proportion of individuals from the original 1982 cohort with depression data in 2012–13, according to selected characteristics. Table describing losses to follow-up, and differences between baseline and the 30 years follow-up. (DOCX 13 kb) Additional file 3: Table S2. Association between self-reported skin color, and African ancestry, major depression and discrimination. We categorized the variable skin color in three different ways for this table, in order to show the association with depression and discrimination, using the original variable with three categories, or when joining the Pardo category with black or white individuals. (DOCX 13 kb) Additional file 4: Table S3. Mediation analysis of the association between African ancestry and major depression in individuals in the highest tertile of socioeconomic position (SEP). Table describing the exact measures of association with confidence intervals for the total, natural direct, and natural indirect effect, product of the G-computation analysis. Only for those individuals in the highest tertile of SEP. (DOCX 12 kb) Additional file 5: Data afr dep bmc.csv. Data base “data afr dep bmc.csv”. We included our database in the csv format, in order to follow the norms of availability of data and material. (CSV 203 kb)

## Abbreviations

CI: Confidence interval
HGDP: Human genome diversity project
IQR: Interquartile ranges
MINI: Mini international neuropsychiatric interview
NDE: Natural direct effect
NIE: Natural indirect effect
PR: Prevalence ratio
SD: Standard deviation
SEP: Socioeconomic position
SNP: Single nucleotide polymorphism
