# Supplementary material for: Genomic ancestry and the social pathways leading to major depression in adulthood: the mediating effect of socioeconomic position and discrimination
Source: BMC Psychiatry. 2016 Sep 5;16(1):308. doi: 10.1186/s12888-016-1015-2 (PMC5011949; doi:10.1186/s12888-016-1015-2)
Supplement: Additional file 1: — Details for socioeconomic and discrimination variables. Here we present more details on how socioeconomic variables, achieved schooling, family income, and household assets index score, were collected and stratified during the last follow-up (30 years). In addition, we explain how this were joined together using a factorial analysis. Details, labels and other information regarding Database “data afr dep bmc.csv”. We included the file “data afr dep bmc.csv”, in order to follow the norms of availability of data and materials. In this section of our Additional file 1, we have included details on the variables in this database. This includes categorization of the variables, labels, and other relevant information. (DOCX 15 kb) [file 12888_2016_1015_MOESM1_ESM.docx]

**Details for socioeconomic and discrimination variables**.

At the mean age of 30.2 years, the following socioeconomic variables were measured:

- Achieved schooling: the highest school grade successfully completed by the individual.
- Household assets index score, based on the individuals and family belongings and possessions, including: number of TVs at home, having a radio, number of cars, having a vacuum cleaner, having a cleaning maid, washing machine, DVD player, number of computers, video games, broadband internet access, microwave oven, refrigerator, number of bathrooms and characteristics, number of bedrooms, and house material.
- Family income: total income, in Brazilian reais, earned by family members in the last month. We asked to the cohort members, how much money did they earned during the last month, from work or any other activities, as well as all other forms of income earned by other family members who lived with him/her. This included money from a retirement plan; any government cash transfer program, or any other possible income.

We used exploratory factorial analysis to create a single variable with these three components of SEP. In addition, this variable was categorized into three tertiles, the first tertile represented the lowest SEP and the third the highest.

We assessed perception of discrimination using the following questions:

- During the last year, did you feel discriminated in any place or by anyone, because of your:

1. skin color;
2. religion or beliefs; and/or
3. for being rich or poor?

**Database “data afr dep bmc.csv” details.**

| Variable | Label | Categories |
| --- | --- | --- |
| psex | Individual sex | 1 men  2 female |
| prenda | Number of minimum salaries the Family earned during the last month | 1: One or less minimum salaries  2: >1-3 minimum salaries  3: >3-6 minimum salaries  4: >6-10 minimum salaries  5: Ten or more minimum salaries |
| pescmaeg | Maternal schooling | 0: zero to four years  1: 5 to 8 years  2: 9 to 11 years  3: 12 or more years |
| momest1982 | Maternal marital status | 1: married or live with partner  2: not married |
| prisco | Gestational risk factors | 1: none  2: GD/Toxemia/HTA  3: previous mischarge or abortion  4: mischarge threat in this pregnancy  5: any other risk |
| momsmk1982 | Pregnancy maternal smoking | 1: no smoking  2: smoking |
| pidmaegr | Maternal age at birth | 1: <20 years  2: 20-29 years  3: 30 or more years |
| dj2 | Skin color/race discrimination | 0: no  1: yes |
| dj3 | Religion/beliefs discrimination | 0: no  1: yes |
| dj5 | discrimination for been rich or poor | 0: no  1: yes |
| gest1982 | Number of gestations | 1: 1 previous gestation  2: 2 previous gestations  3: 3 previous gestations  4: 4 or more previous gestations |
| part1982 | Type of delivery | 0: C-section  1: vaginal |
| esccat | Schooling at 30 years | 0: zero to four years  1: 5 to 8 years  2: 9 to 11 years  3: 12 or more years |
| abep | Assets index in categories | 0: A/B  1: C  2: D/E |
| rendater | Family income at 30 years in tertiles | 0: lowest tertile  1: middle tertile  2: highest tertile |
| eurq | European ancestry | 0: <5%  1: 5-30%  2: more than 30% |
| afrq | African ancestry | 0: <5%  1: 5-30%  2: more than 30% |
| dvdepman | Major depression at 30 years | 0: no  1: yes |
| cor3 | Self-reported skin color | 1: white  2: pardo  3: black |
