# Supplementary material for: Genomic ancestry and the social pathways leading to major depression in adulthood: the mediating effect of socioeconomic position and discrimination
Source: BMC Psychiatry. 2016 Sep 5;16(1):308. doi: 10.1186/s12888-016-1015-2 (PMC5011949; doi:10.1186/s12888-016-1015-2)
Supplement: Additional file 2: Table S1. — Proportion of individuals from the original 1982 cohort with depression data in 2012–13, according to selected characteristics. Table describing losses to follow-up, and differences between baseline and the 30 years follow-up. (DOCX 13 kb) [file 12888_2016_1015_MOESM2_ESM.docx]

**Additional file 2: Table S1. Proportion of individuals from the original 1982 cohort with depression data in 2012-13, according to selected characteristics**

|  | **Original cohort (number)** | **Followed at 30 years (%)** | **Followed at 30 years with depression data (%)** | **p-value*** |
| --- | --- | --- | --- | --- |
| Gender |  |  |  | 0.167 |
| Male | 3037 | 65.2 | 96.9 |  |
| Female | 2876 | 71.1 | 96.1 |  |
| Skin color (mother) | |  |  |  |
| White | 4851 | 68.0 | 96.6 | 0.251 |
| Non-white | 1060 | 71.3 | 97.9 |  |
| Skin color (self-reported)** | | |  | 0.193 |
| White | 3238 | 79.7 | 92.6 |  |
| Pardo | 385 | 80.0 | 90.6 |  |
| Black | 673 | 81.9 | 90.7 |  |
| Family Income at birth (Minimum wages) | | | | 0.156 |
| ≤1 | 1288 | 66.1 | 95.3 |  |
| >1-3 | 2789 | 70.4 | 97.0 |  |
| >3-6 | 1091 | 69.3 | 95.8 |  |
| >6-10 | 382 | 61.3 | 97.3 |  |
| ≥10 | 335 | 60.3 | 98.0 |  |
| Maternal schooling | |  |  | 0.768 |
| 0-4 | 1960 | 68.0 | 96.2 |  |
| 5-8 | 2454 | 70.5 | 96.6 |  |
| 9-11 | 654 | 66.1 | 97.3 |  |
| ≥12 | 839 | 62.8 | 96.3 |  |

*Chi-squared p-value for heterogeneity between those followed at 30 years and those with mental health data

**Self-reported skin color was collected only at 23 years. Therefore, the column “original cohort (number)” for this case, refers to the follow-up at 23 years.
