# Supplementary material for: Genomic ancestry and the social pathways leading to major depression in adulthood: the mediating effect of socioeconomic position and discrimination
Source: BMC Psychiatry. 2016 Sep 5;16(1):308. doi: 10.1186/s12888-016-1015-2 (PMC5011949; doi:10.1186/s12888-016-1015-2)
Supplement: Additional file 3: Table S2. — Association between self-reported skin color, and African ancestry, major depression and discrimination. We categorized the variable skin color in three different ways for this table, in order to show the association with depression and discrimination, using the original variable with three categories, or when joining the Pardo category with black or white individuals. (DOCX 13 kb) [file 12888_2016_1015_MOESM3_ESM.docx]

**Additional file 3: Table S2. Association between self-reported skin color, and African ancestry, major depression and discrimination.**

|  |  | **Total N** | **African Ancestry** | | | | **Major depression** | | **Discrimination** | | | | | |
| --- | --- | --- | --- | --- | --- | --- | --- | --- | --- | --- | --- | --- | --- | --- |
|  |  |  | **0-5%** | **>5-30%** | **>30-90%** | **p** | **Prevalence** | **p** | **Skin color** | **p** | **Religion** | **p** | **Socioeconomic** | **p** |
| skin color | Black + Pardo | 813 | 4.30% | 21.00% | 74.70% | <0.001 | 9.40% | 0.07 | 16.90% | <0.001 | 10.00% | 0.002 | 10.10% | <0.001 |
|  | White | 2455 | 52.50% | 45.60% | 2.00% |  | 7.40% |  | 2.00% |  | 6.70% |  | 5.70% |  |
| skin color | Black | 516 | 0.80% | 6.70% | 92.50% | <0.001 | 9.30% | 0.193 | 21.20% | <0.001 | 9.50% | 0.007 | 10.30% | <0.001 |
|  | Pardo | 297 | 10.40% | 46.50% | 43.10% |  | 9.40% |  | 9.50% |  | 10.80% |  | 9.80% |  |
|  | White | 2455 | 52.50% | 45.60% | 2.00% |  | 7.40% |  | 2.00% |  | 6.70% |  | 5.70% |  |
| skin color | Black | 516 | 0.80% | 6.70% | 92.50% | <0.001 | 9.30% | 0.186 | 21.20% | <0.001 | 9.50% | 0.056 | 10.30% | 0.001 |
|  | White + Pardo | 2751 | 47.90% | 45.70% | 6.40% |  | 7.60% |  | 2.80% |  | 7.10% |  | 6.20% |  |
