# Supplementary material for: Genomic ancestry and the social pathways leading to major depression in adulthood: the mediating effect of socioeconomic position and discrimination
Source: BMC Psychiatry. 2016 Sep 5;16(1):308. doi: 10.1186/s12888-016-1015-2 (PMC5011949; doi:10.1186/s12888-016-1015-2)
Supplement: Additional file 4: Table S3. — Mediation analysis of the association between African ancestry and major depression in individuals in the highest tertile of socioeconomic position (SEP). Table describing the exact measures of association with confidence intervals for the total, natural direct, and natural indirect effect, product of the G-computation analysis. Only for those individuals in the highest tertile of SEP. (DOCX 12 kb) [file 12888_2016_1015_MOESM4_ESM.docx]

**Additional file 4: Table S3. Mediation analysis of the association between African ancestry and major depression in individuals in the highest tertile of socioeconomic position (SEP).**

| **G-Computation estimate (95%CI)** | |  |
| --- | --- | --- |
| **Natural direct effect** | **Natural indirect effect** | **Mediated effect** |
| *Mediated by skin color Discrimination* | |  |
| 0.0068 (-0.0168; 0.0303) | 0.0352 (0.0128; 0.0578) | 83.9% |
| *Mediated by religion/beliefs Discrimination* | |  |
| 0.0217 (-0.0036; 0.0469) | 0.0081 (-0.0154; 0.0317) | 27.3% |
| *Mediated by rich or poor Discrimination* | |  |
| 0.0203 (-0.0038; 0.0444) | 0.0068 (-0.0155; 0.0291) | *25%* |
| *Adjusted for base confounder gender, and post confounders schooling and income at 30 years. CI=Confidence Interval | | |
